# Factors associated with the fertility of the helminth parasite *Ascaris lumbricoides*: an exploratory study in a rural Venezuelan community

**DOI:** 10.1016/j.fawpar.2026.e00363

**Published:** 2026-09-23

**Authors:** Nelson Escalona, Lapo Mughini-Gras, Eva Lorenz, Luis Sequera, Ivan Sequera, Ruth Serrano, Renzo Nino Incani

**Affiliations:** a Institute of Medical Biostatistics, Epidemiology and Informatics, University Medical Center of the Johannes Gutenberg-University Mainz, Germany; b Institute for Risk Assessment Sciences (IRAS), Utrecht University, Utrecht, the Netherlands; c Center for Infectious Diseases Control, National Institute for Public Health and the Environment (RIVM), Bilthoven, the Netherlands; d Department of Infectious Diseases Epidemiology, Bernhard Nocht Institute for Tropical Medicine (BNITM), Hamburg, Germany; e Department of Parasitology, Faculty of Health Sciences, Universidad de Carabobo, Valencia, Venezuela

**Keywords:** *Ascaris lumbricoides*, Fertility, Worm biometrics, Crowding effect, Venezuela

## Abstract

The helminth parasite *Ascaris lumbricoides* remains a major global health burden, yet factors influencing its fertility and infection intensity are not fully understood. This cross-sectional study explored associations between host characteristics, worm biometrics, and parasite reproductive output in a rural Venezuelan community. Complete fecal evacuations were collected from 128 participants (57% of consenting population) across 45 houses. Following descriptive analyses of worm-related variables (count, length, weight, sex, male-to-female ratio, maturity), egg-related variables (eggs per gr of feces [EPG], fertile and infertile EPG, infertile-to-fertile ratio), and host characteristics (gender, age, household), both univariate methods and multivariable regression analyses were performed to assess associations. Worm biometrics showed non-significant associations with infection type (unisexual vs. bisexual), although male worms in unisexual infections tended to be heavier. A non-significant crowding effect was observed, with higher worm counts often linked to lower average worm length and weight. Worm length and weight were strongly correlated with egg production, though this relationship weakened in bisexual infections, likely due to reduced female fecundity. Male worm presence appeared to be the main determinant of egg fertility, with higher male-to-female ratios showing borderline significant association with lower ratios of unfertilized to fertilized eggs. Host age and household clustering were associated with worm count, whereas host gender was not. These findings support key biological principles of *A. lumbricoides*, including density-dependent crowding effects and male worm presence in egg fertility. The study also highlights the complexity of worm-specific and host-related factors influencing infection intensity and fecundity, providing insights for future research and more targeted deworming strategies.

## Highlights

• ∼40% of *A. lumbricoides* infections may be undetectable due to unisex/immature worms.
• Worm biometrics differed between unisexual and bisexual infections.
• Male worms in unisexual infections tended to be heavier.
• Worm length and weight correlated with egg production, but estimates were imprecise.
• Host age, household clustering, and sex ratio were associated with infection patterns.

## Graphical abstract

**Figure:**
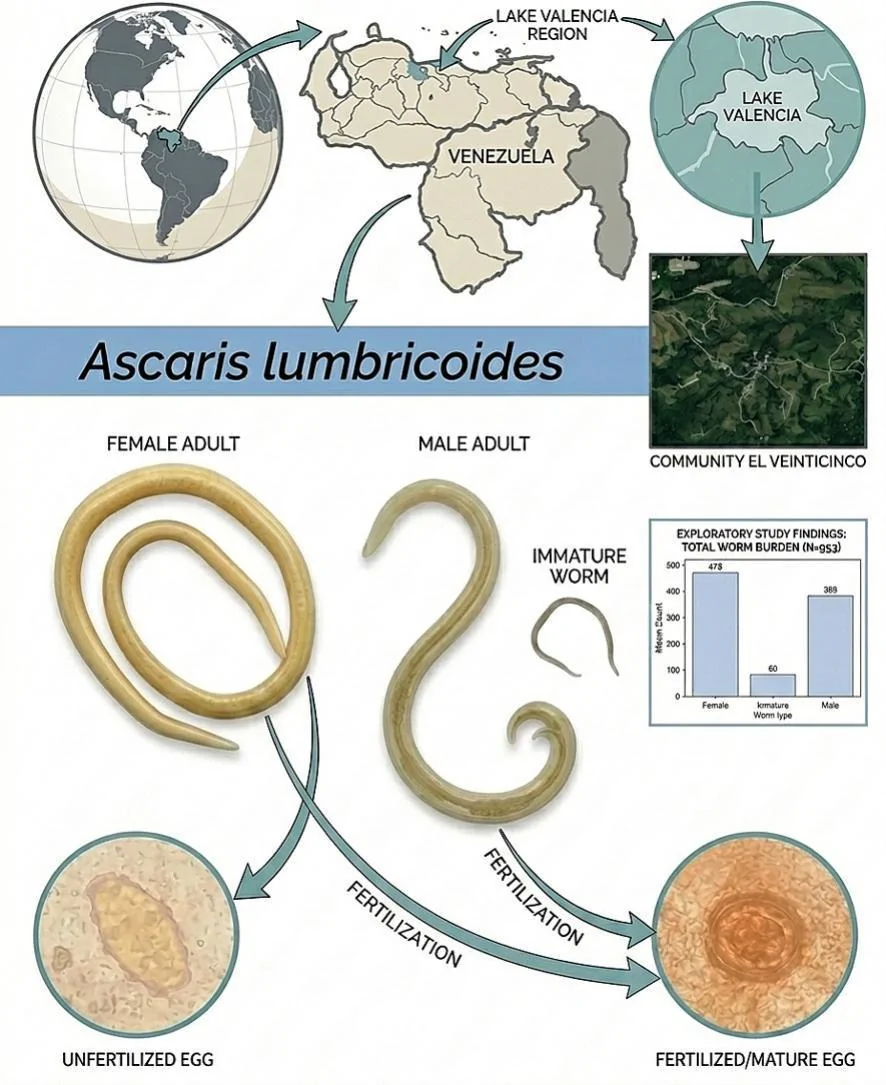

## 1 Introduction

*Ascaris lumbricoides*, a soil-transmitted helminth, is the most prevalent human parasitic nematode worldwide, infecting an estimated 732 (682–782) million people, primarily in tropical and subtropical regions with inadequate sanitation (Holland et al., 2022). Despite global efforts to control these infections, they remain a significant public health burden, particularly affecting children in low- and middle-income countries. Indeed, infection intensity correlates strongly with adverse health outcomes, such as malnutrition, growth stunting, and impaired cognitive development, while also being the main driver of environmental contamination and reinfection dynamics. Nevertheless, infection intensity is often neglected, and studies frequently favor prevalence as a key epidemiological measure (Hall et al., 2008; Walker et al., 2013; Holland et al., 2022; Veesenmeyer, 2022).

The parasite life cycle involves ingestion of embryonated eggs, larval migration through the lungs, and maturation to adult worms in the host small intestine (Betson et al., 2025). A key aspect of the parasite's success is its high reproductive capacity, with female worms capable of producing over 200,000 eggs per day (Betson et al., 2025). The number of eggs shed by a host is a critical measure for both diagnostic and epidemiological purposes, often used as a proxy for the intensity of infection (Walker et al., 2009). However, the relationship between worm burden and egg output is complex, influenced by a variety of factors (Walker et al., 2009). The fertility of female worms is contingent upon cohabitation with male worms, but the dynamics of this process, including the effects of worm size, worm density (crowding), and sex ratio, are not fully understood. Understanding these factors is crucial for accurate interpretation of parasitological surveys and for predicting the impact of control interventions.

This exploratory study aims to address these knowledge gaps by investigating the factors associated with the fertility of *A. lumbricoides* worms. Using comprehensive data collected during a 2009 cross-sectional study of 224 individuals in a rural community in Carabobo State, Venezuela (Incani et al., 2021), we investigated associations between worm characteristics, host-related factors, and parasite reproductive output. Specifically, we examined the associations between worm size and type of infection (unisexual vs. bisexual), the crowding effect, and the impact of the male-to-female worm ratio on egg output. By exploring these relationships, this study sought to enhance our understanding of *A. lumbricoides* population dynamics, generate hypotheses for future studies, and contribute to more effective infection control strategies.

## 2 Materials and methods

### 2.1 Study area and population

This cross-sectional study was conducted in “Caserio El 25,” a rural community in Carabobo State, Venezuela, situated at 67°52′58“ W and 10°02’30” N. The area has a tropical, humid climate, with an average temperature of 30 °C and 75% humidity, providing an environment conducive to soil-transmitted helminth transmission. The study population was drawn from a previously published epidemiological survey of 224 participants designed to characterize reinfection with *A. lumbricoides* following anthelmintic treatment (Incani et al., 2022). For the present study, we performed new analyses of the participant- and worm-level measurements collected during the original 2009 survey. These data remain relevant because core biological relationships in parasite ecology are expected to persist despite potential temporal changes in infection prevalence since data collection. Exclusion criteria for participants included pregnancy, severe illness, or absence from home during the treatment period. Ethical approval was obtained from the appropriate institutional review board, and all participants provided informed consent. Further details on the study setting can be found elsewhere (Incani et al., 2021, Incani et al., 2022).

### 2.2 Parasite and host data collection

Following a baseline fecal examination, all participants received a single oral dose of an anthelminthic drug (Pyrantel pamoate^R^). To ensure complete worm expulsion and collection, participants were instructed to collect all fecal samples over a 48-h period post-treatment. These samples were sieved in the field and the recovered *A. lumbricoides* worms were washed, counted, and preserved in 10% formaldehyde in individual containers for transportation to the laboratory. In the laboratory, each worm was individually sexed based on its tail morphology, and its length (cm) and weight (g) were measured. Worms smaller and thinner than average, without visible sexual differentiation, and corresponding to pre-reproductive juvenile stages, were considered immature. In addition to worm collection, pre-treatment fecal samples were analyzed using the Merthiolate-Iodine-Formaldehyde (MIF) fixation method to quantify eggs per gram (EPG) (Incani et al., 2022). These counts were used to determine the total number of eggs, as well as the fertilized and unfertilized egg ratios. A standardized questionnaire was used to collect demographic data from participants, including age, gender, and household location to evaluate potential clustering effects.

### 2.3 Statistical analysis

Statistical analyses were conducted using SAS® OnDemand for Academics. Descriptive statistics were used to summarize worm-related variables (total count, length, weight, sex, male-to-female ratio, maturity), egg-related variables (total EPG, fertile and infertile EPG, infertile-to-fertile ratio), and host characteristics (age, gender, household). The distribution of continuous variables was examined using histograms, QQ-plots, and summary measures. Given the limited sample size, analyses were primarily descriptive. Subgroup analyses, particularly those comparing unisexual (*n* = 28) and bisexual (*n* = 49) infections and analyses stratified by sex, involved small sample sizes and should therefore be interpreted with caution.

Associations between continuous variables were assessed using Spearman's correlation coefficients. Group comparisons were performed using *t*-tests or ANOVA when variables were approximately normally distributed. The relationships between worm size, worm burden, and infection type (unisexual vs. bisexual) were evaluated using *t*-tests and logistic regression models. The potential crowding effect on worm size was examined using linear regression.

Because egg counts (EPG and its components) are discrete and typically right-skewed, count regression models were applied. Negative binomial regression was selected over Poisson models due to overdispersion. These models were used to assess the association between EPG and the number, length, and weight of female worms, as well as to compare fertility patterns between unisexual and bisexual infections. The association between the male-to-female worm ratio and the unfertilized-to-fertilized egg ratio was also examined using negative binomial regression.

Mixed-effects models with random intercepts for households were fitted to evaluate host-related factors (age, gender) while accounting for the hierarchical structure of the data and the known clustering of infections within homes. Logistic regression was used for categorical outcomes, such as infection type.

A significance level of *p* < 0.05 was used for all hypothesis tests, and effect sizes with 95% confidence intervals (95% CI) were reported. As the study was exploratory, no correction for multiple testing was applied. Consequently, the reported *p*-values should be interpreted with caution because of the increased risk of Type I error. All models were evaluated using residual diagnostics and Akaike information criterion (AIC). Fixed effects included worm-related variables (worm count, worm biometrics, sex ratios) and host characteristics (age, gender), depending on the model.

Missing data were minimal across variables (< 5% for all predictors and outcomes). As most missing values were due to technical factors (e.g., damaged or unmeasurable worms) and were unlikely to be related to host characteristics or infection status, complete-case analysis was conducted. The number of observations included in each model is reported in the corresponding tables.

No a priori sample size calculation or power analysis was performed, as the study was exploratory and enrolment was limited by available data, resources, and eligibility criteria. The adequacy of the sample size was assessed by examining the width of reported confidence intervals.

## 3 Results

### 3.1 Study population characteristics and general findings

Of the approximately 470 inhabitants in the community, 224 individuals (48%) living in 55 of 85 houses (65%) provided informed consent and participated in the initial epidemiological study. A subset of 128 participants (57%) across 45 houses (82%) provided at least one full fecal sample within the 48-h post-treatment collection period. The remaining individuals were excluded due to non-compliance with the collection protocol or other exclusion criteria. The study group was composed of 58 males (45%) and 70 females (55%).

Among the 128 participants with complete fecal samples, 89 (70%) were positive for *A. lumbricoides* worms and/or eggs; these were 43 males (48%) and 46 females (52%). Worms were detected in 80 samples, and eggs in 65 samples. Further analyses were therefore carried out on those 80 samples with detectable worms. Participants with no detectable eggs pre-treatment had higher odds of not expelling worms, demonstrating strong agreement between pre-treatment egg counts and worm expulsion (odds ratio [OR] = 10.0, 95% CI 4.24–24.09).

The study population exhibited substantial variability in infection intensity, reflected in both worm burden and egg production. A total of 953 worms were recovered from 89 infected individuals, with females (*n* = 478, mean = 5, standard deviation [SD] = 10, range = 0–71) being more numerous than males (*n* = 386, mean = 4, SD = 8, range = 0–51) and immature worms (*n* = 89, mean = 1, SD = 3, range = 0–27). Egg production was also considerable. Aggregated EPG values indicated that fertile eggs accounted for approximately 1.48 × 10^6^ eggs (mean = 16,601, SD = 29,452, range = 0–148,710), while infertile eggs contributed 2.85 × 10^5^ eggs (mean = 3211, SD = 13,959, range = 0–125,000), yielding a combined total of 1.76 × 10^6^ eggs across the population (Supplementary Figs. S1 and S2).

Participants aged 6 to 10 years constituted the largest age group in the study (23%), while those aged 11 to 15 years represented the smallest proportion (10%). Among infected individuals, the age distribution was similar, though children under 6 years expelled the highest number of worms (54%), while individuals aged 16–25 years had the lowest worm burden.

### 3.2 Worm size, infection type, and crowding effect

#### 3.2.1 Worm characteristics and infection type

The expelled worms showed expected sexual dimorphism in their biometrics. The mean weight for all collected worms (*n* = 953) was 2.22 g (SD = 1.72, range 0–8 g), and the mean length was 18.62 cm (SD = 6.15, range 2–33.8 cm). Female worms (*n* = 478) were larger than male worms (*n* = 386) in both weight (mean 3.4 g vs. 1.2 g) and length (mean 22.7 cm vs. 16.2 cm). Approximately 90% of worms exceeded 15 cm, indicating that most were mature adults.

Among the 80 infected participants with worms, bisexual infections (presence of both male and female worms) were the most common (61.3%), followed by unisexual infections (35%), of which 22.5% were female-only and 12.5% were male-only, and 3.7% were immature non sexually defined worms (Fig. 1).

**Fig. 1:**
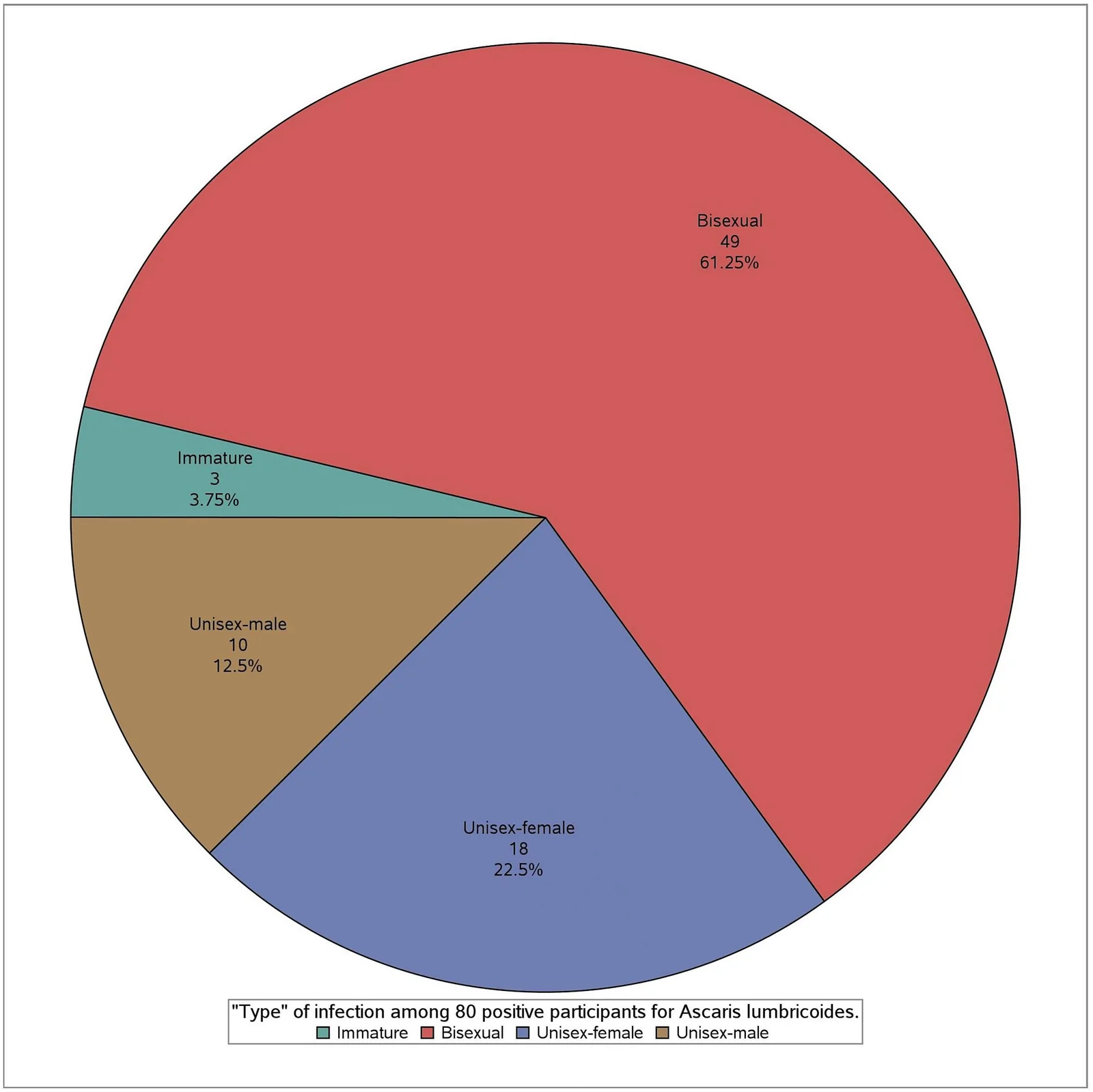
Distribution of *A. lumbricoides* infections by infection type. (*n* = 80).

A two-sample *t*-test revealed no significant difference in the mean average female worm length between unisexual-female and bisexual infections (*p* = 0.22). Similarly, a logistic regression model, with bisexual infection as the reference, showed that average female worm length was not a significant predictor of infection type (OR = 0.95, *p* = 0.55, 95% CI 0.82–1.12, Fig. 2). A one-unit increase in average female worm length was associated with a non-significant 5% decrease in the odds of a unisexual-female infection. Adjusting for female worm count did not change this result (OR = 0.95, *p* = 0.55, 95% CI 0.815–1.116). Similar findings were observed for male worms (*p* = 0.33), where the average male worm length was not a significant predictor of unisexual-male infections (OR = 0.93, *p* = 0.70, 95% CI 0.65–1.33, Fig. 3).

**Fig. 2:**
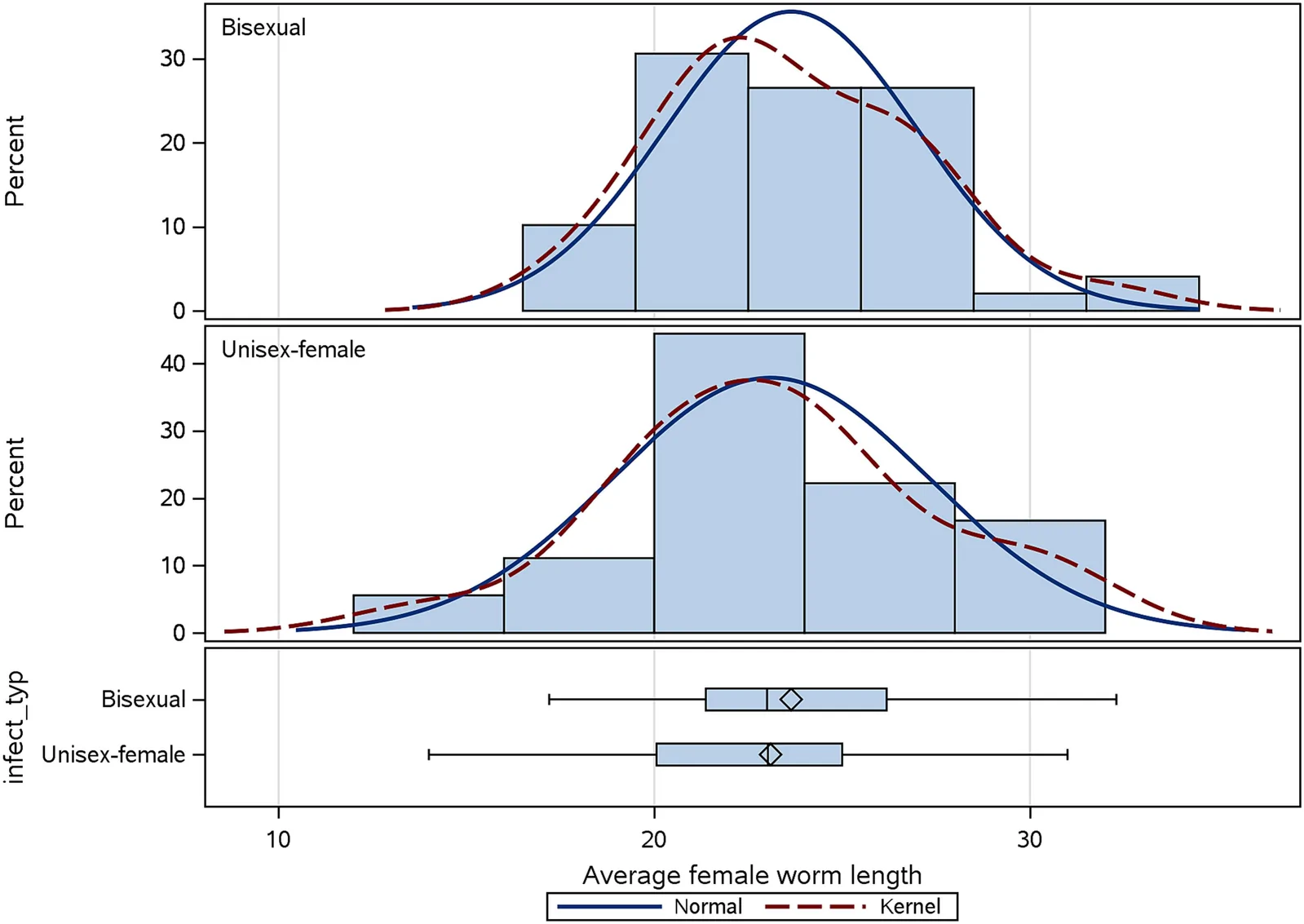
Comparison of the frequency distributions of mean female *A. lumbricoides* worm length between unisexual female (*n* = 18) and bisexual (*n* = 49) infections. Normal and kernel density curves are overlaid on the distributions.

**Fig. 3:**
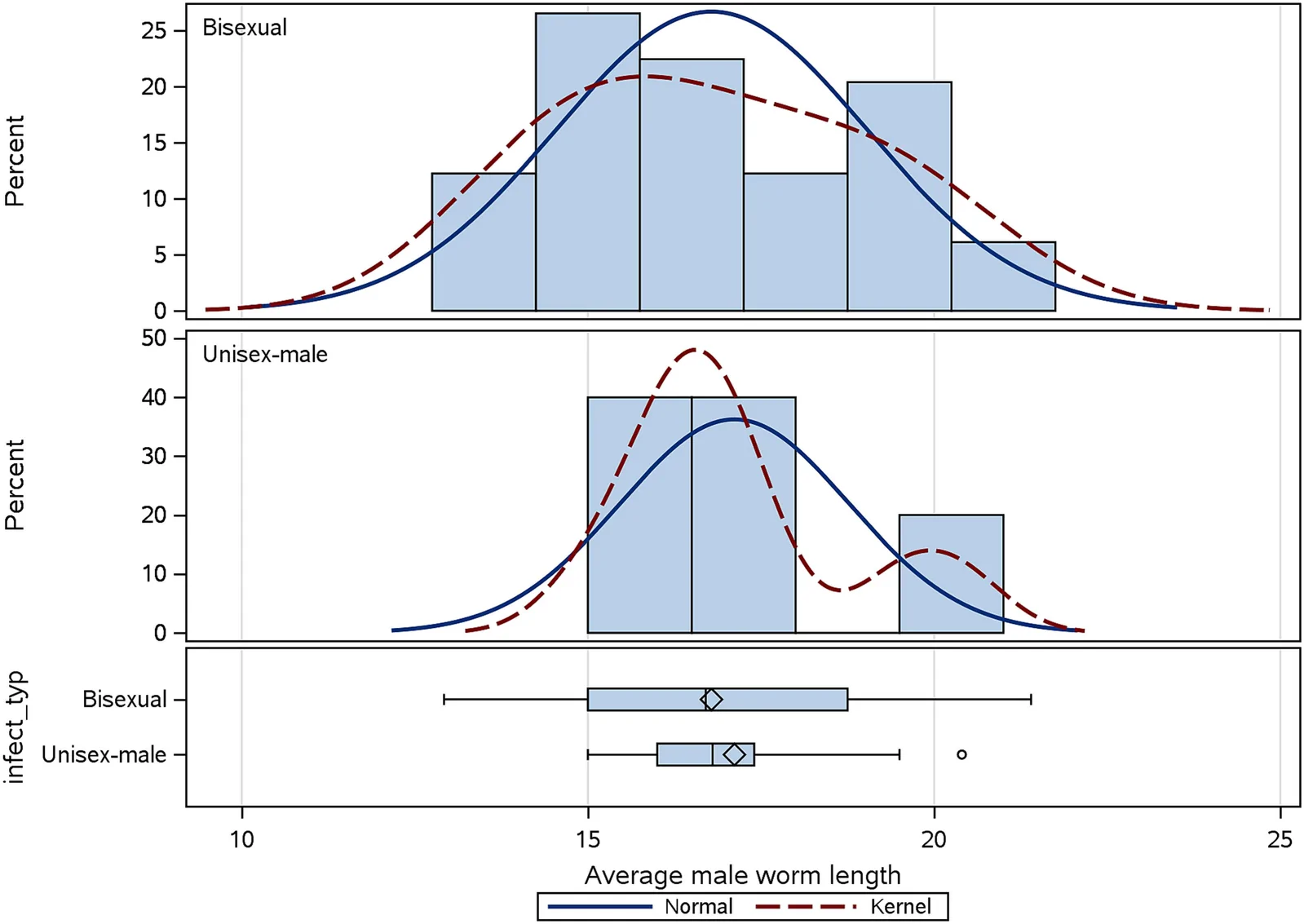
Comparison of the frequency distributions of mean male *A. lumbricoides* worm length between unisexual male (*n* = 10) and bisexual (*n* = 49) infections. Normal and kernel density curves are overlaid on the distributions.

Average worm weight also showed no significant association with infection type. For female worms, a *t*-test showed no significant difference between the two infection groups (*p* = 0.56). A logistic regression model confirmed this, with average female worm weight not a significant predictor (OR = 0.81, *p* = 0.32, 95% CI 0.54–1.22, Fig. 4). For male worms, the *t*-test (*p* = 0.71) and logistic regression (OR = 1.86, *p* = 0.48, 95% CI 0.33–10.43, Fig. 5) also showed no significant association between average weight and infection type.

**Fig. 4:**
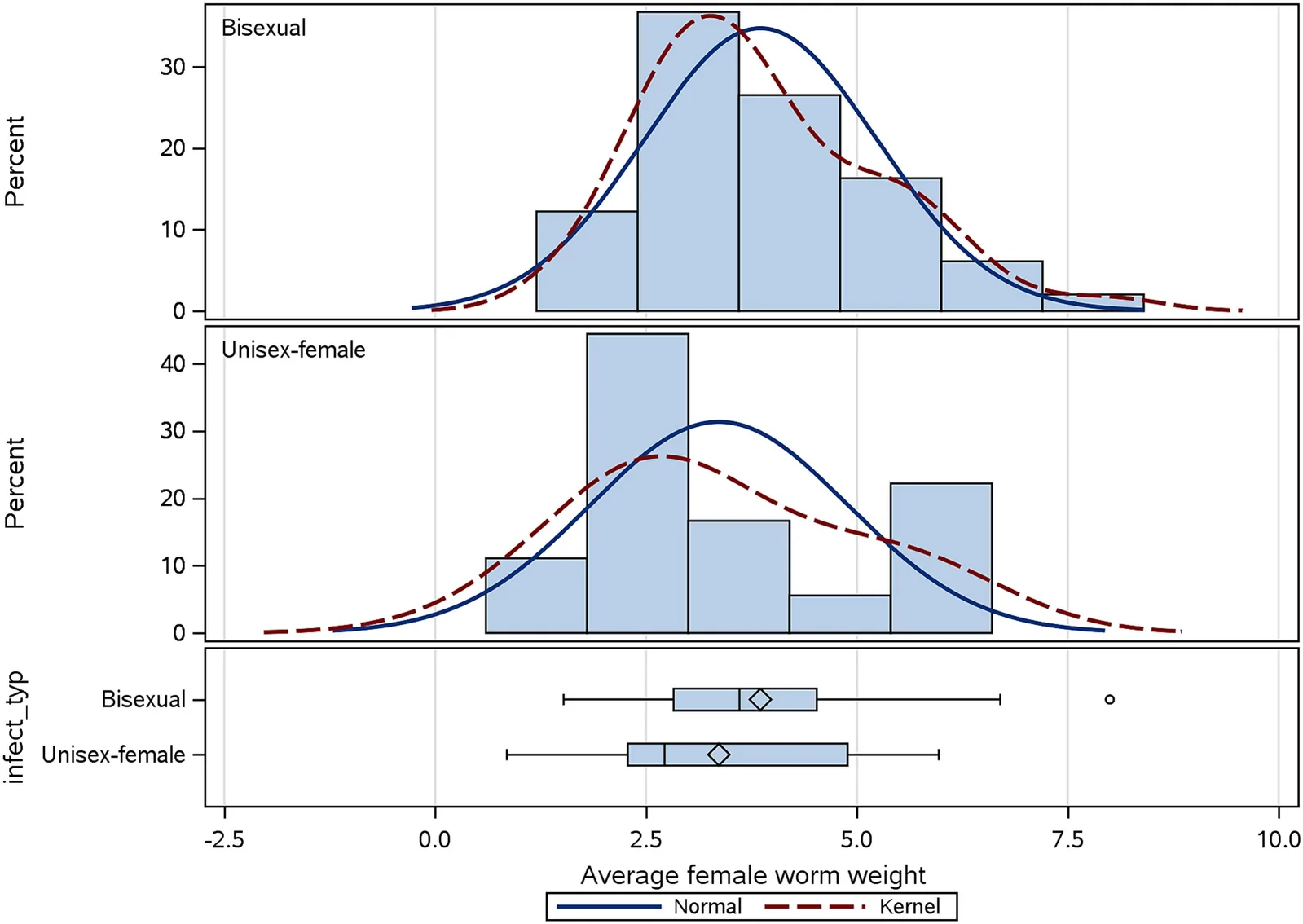
Comparison of the frequency distributions of mean female *A. lumbricoides* worm weight between unisexual female (*n* = 18) and bisexual (*n* = 49) infections. Normal and kernel density curves are overlaid on the distributions.

**Fig. 5:**
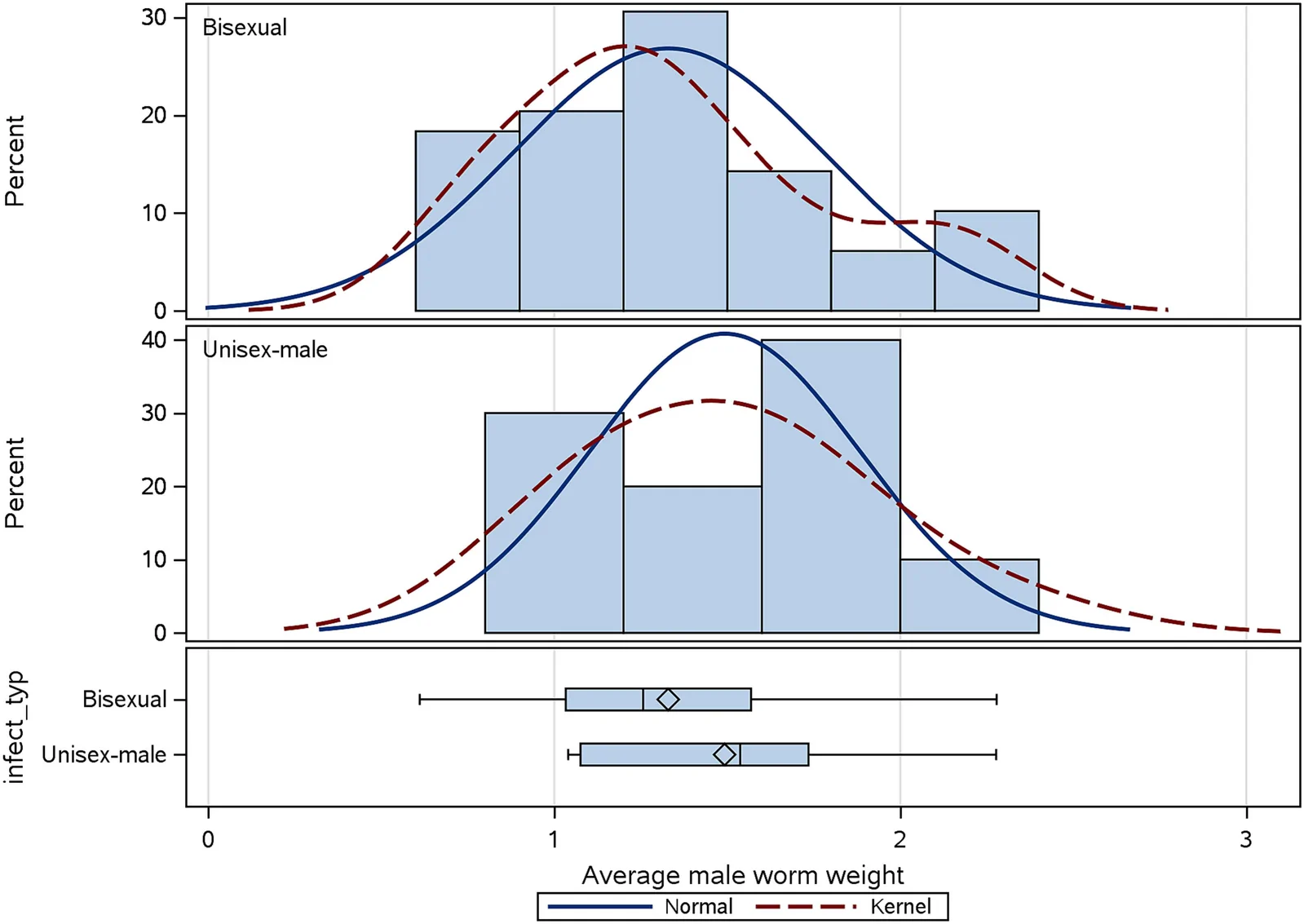
Comparison of the frequency distributions of mean male *A. lumbricoides* worm weight between unisexual male (*n* = 10) and bisexual (*n* = 49) infections. Normal and kernel density curves are overlaid on the distributions.

#### 3.2.2 Worm size and crowding effect

A statistically non-significant negative association was observed between total worm count and average worm length, consistent with the crowding effect. Linear regression analysis for female worms indicated that for every one-unit increase in worm count, the average female worm length was associated with a reduction of 0.02 cm (*p* = 0.41). For male worms, a similar trend was observed, with an association of a 0.02 cm reduction per additional worm (*p* = 0.07, Table 1). Similar trends were observed for worm weight. A linear regression model for female worms suggested a reduction of 0.01 g in average weight for every additional worm (*p* = 0.39). Male worms also showed a similar trend, with a reduction of 0.005 g in average weight per additional worm (*p* = 0.06, Table 1).

**Table 1:** Multiple linear regression results of *A. lumbricoides* infection (*n* = 49).

Model	Parameter	Estimate	Std. Error	t-value	*p*-value	Lower 95% CI	Upper 95% CI
Average male length vs. total worm count	Intercept	17.1964	0.3334	51.57		16.5287	17.8641
	Total worms	−0.0229	0.0122	−1.87	0.0668	−0.0474	0.0016
Average male weight vs. total worm count	Intercept	1.4324	0.0679	21.07		1.2962	1.5685
	Total worms	−0.0048	0.0025	−1.94	0.0572	−0.0098	0.0001
Male–female ratio vs. average female length	Intercept	−1.1666	1.3057	−0.89	0.3761	−3.7933	1.4600
	Average female length	0.1053	0.0546	1.93	0.0600	−0.0046	0.2154
Male–female ratio vs. average female weight	Intercept	0.3867	0.5476	0.71	0.4836	−0.7150	1.4884
	Average female weight	0.2434	0.1339	1.82	0.0755	−0.0259	0.5128

### 3.3 Worm count and maturity

Analyses consistently indicated no association between worm burden and maturity structure. The observed correlation between the total number of worms per host and the ratio of mature to immature worms was weak and non-significant (Spearman's correlation coefficient [SCC] = 0.15, *p* = 0.19). Likewise, the negative binomial regression model showed non-significant effect of increasing worm count on the maturity ratio: a one-unit increase in worm burden corresponded to only a non-significant 0.12% change in the proportion of mature worms (incidence rate ratio [IRR] = 1.0012, 95% CI 0.99–1.01, *p* = 0.75). These results suggest that higher worm counts do not correspond to higher proportions of mature worms, indicating that maturation is likely determined by factors other than crowding.

### 3.4 Egg production and worm characteristics

#### 3.4.1 Female-only infections

In female-only (unisexual) infections, the number of female worms showed a moderate positive correlation with EPG (SCC = 0.62, *p* = 0.005). However, the corresponding negative binomial regression estimate was highly imprecise (spanning from negligible to a 15-fold change in EPG per additional female worm): each additional female worm was associated with an expected 1.37-fold change in EPG (95% CI 0.12–15.12, *p* = 0.80), indicating that there is insufficient data to support any definitive conclusions about this association.

The total length of female worms per host exhibited a very strong positive correlation with EPG (SCC = 0.84, *p* < 0.0001). Yet, the regression model produced a non-significant estimate, with a 1.06-fold expected change in EPG per additional centimeter of worm length (95% CI 0.92–1.21, *p* = 0.41). Similarly, the total weight of female worms was strongly correlated with EPG (SCC = 0.85, *p* < 0.0001), but the regression coefficient showed wide uncertainty, suggesting a 2.35-fold change in EPG per unit increase in worm weight (95% CI 0.96–5.74, *p* = 0.06), which again did not reach statistical significance.

Taken together, these analyses show that although simple correlations suggest strong positive relationships between female worm metrics and EPG, the regression models, affected by small sample size and wide confidence intervals, provided no statistically significant evidence for these associations. The severe imprecision of the model estimates means that the true effects could plausibly range from negligible to large, and therefore any interpretation must remain cautious.

#### 3.4.2 Bisexual infections

In bisexual infections, the number of female worms showed a positive correlation with EPG (SCC = 0.64, *p* < 0.0001), similar in magnitude to that observed in unisexual infections. However, the negative binomial regression estimate was not statistically significant and displayed considerable uncertainty, with each additional female worm associated with an expected 1.06-fold change in EPG (95% CI 0.96–1.17, *p* = 0.23, Table 2), which indicates that this association is imprecise and not reliably supported.

**Table 2:** Negative binomial regression results for EPG and female worm count in bisexual infections of *A. lumbricoides* (*n* = 49).

Parameter	Estimate	Wald 95% Confidence Limits		Pr > ChiSq
Intercept	9.691	8.599	10.784	<0.001
Female worms	0.060	−0.038	0.159	0.232
Dispersion	4.604	3.251	6.522	

The correlation between total female worm length and EPG was weaker in bisexual infections (SCC = 0.69, *p* < 0.0001) compared with unisexual infections. The corresponding regression estimates again showed no significant effect and very narrow but still uncertain confidence intervals, with a 1.003-fold expected change in EPG per additional centimeter of worm length (95% CI 0.999–1.008, *p* = 0.16). Similarly, total female worm weight was strongly correlated with EPG (SCC = 0.71, *p* < 0.0001), but the regression model indicated only a non-significant 1.03-fold change in EPG per unit increase in worm weight (95% CI 0.99–1.07, *p* = 0.07). Across all three metrics, the contrast between strong correlations and non-significant, imprecise regression estimates suggests that the apparent relationships may be influenced by sample size limitations and shared scaling among worm metrics.

### 3.5 Sex ratio and egg fertility

Mean EPG varied across age groups, with the highest values in children under 6 years and the lowest in adults aged 26–45 years. No significant differences in mean EPG were observed between males and females.

In bisexual infections, a negative correlation was observed between the male-to-female worm ratio and the unfertilized-to-fertilized egg ratio (SCC = −0.28, *p* = 0.05, Fig. 6). The negative binomial regression model suggested that each unit increase in the male-to-female ratio was associated with a 50% reduction in the unfertilized-to-fertilized egg ratio (95% CI 0.24–1.01, *p* = 0.05, Table 3).

**Fig. 6:**
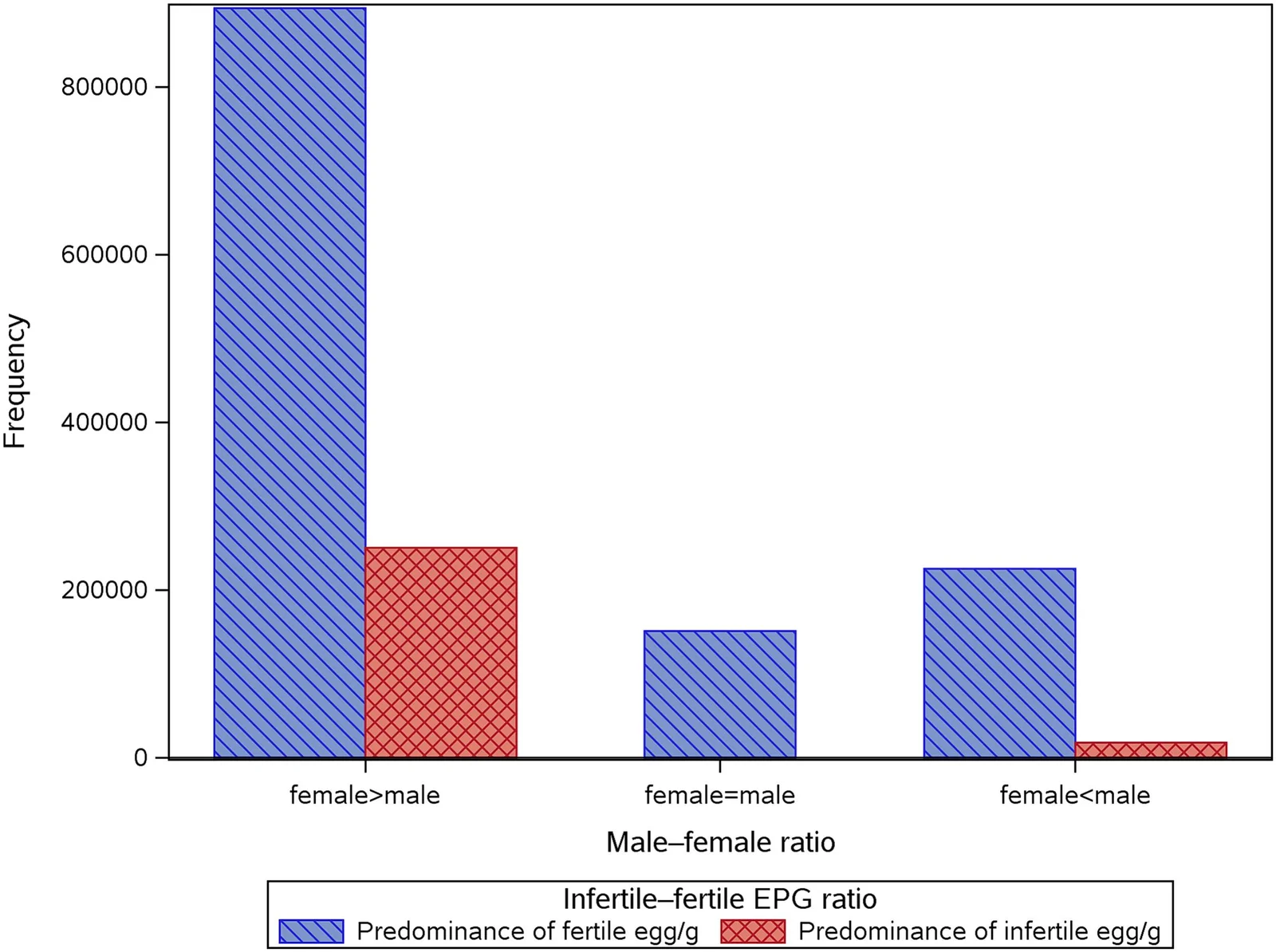
Frequency distribution of egg fertility across male-to-female worm ratio groups in bisexual *A. lumbricoides* infections (*n* = 49).

**Table 3:** Negative binomial regression results for infertile-fertile EPG ratio and male-female worm ratio in *A. lumbricoides* infections.

Parameter	Estimate	Wald 95% Confidence Limits		Pr > ChiSq
Intercept	0.466	−0.331	1.263	0.252
Male-female ratio	−0.698	−1.410	0.013	0.054
Dispersion	1.304	0.566	3.002	

### 3.6 Host factors

A mixed linear model showed a significant association between participant age and worm count (F = 6.88, *p* = 0.01), with worm burden decreasing by 0.17 worms per year of age. This confirms that age is a relevant host factor for infection intensity. Conversely, participant gender showed no significant association with worm count (F = 0.19, *p* = 0.67), suggesting it is not a key factor in determining the number of worms. The inclusion of households as a random effect in the model accounted for the known clustering of infections within homes.

## 4 Discussion

The present study aimed to expand our understanding of *A. lumbricoides* fertility and its associated factors within a rural Venezuelan community, an endemic setting where soil-transmitted helminth infections remain a significant public health issue. The intensity of this worm infection varies markedly across geographical regions, influenced by environmental, climatic, behavioral, and infrastructural factors. Hall and Holland (2000) emphasized that differences in parasite burden between communities often reflect local ecological conditions, including soil composition, humidity, temperature, and sanitation practices. In the present study, the high worm and egg burdens observed are consistent with the tropical, humid environment of the rural Venezuelan community, where conditions favor egg survival and transmission. These findings underscore the importance of contextualizing infection intensity within local ecological and socio-environmental frameworks. Our findings should also be interpreted as exploratory and hypothesis-generating, rather than confirmatory, given the limited sample size and cross-sectional design of the present study. Future research would benefit from larger sample sizes and longitudinal designs, with repeated measurements to improve precision, assess reinfection dynamics, and explore more complex interactions between worm biometrics, host characteristics, and environmental factors.

Our analysis revealed no statistically significant association between the average length or weight of worms and the type of infection (unisexual vs. bisexual). While female worms were, on average, larger than male worms, as is well-documented in the literature, the biometric comparison between worms from unisexual versus bisexual infections did not yield a conclusive result. The non-significant results, where worms appeared somewhat larger in bisexual infections, with the exception of slightly heavier males in unisexual infections, should therefore be interpreted with caution. Given the small sample size and wide variability, it remains uncertain whether these differences represent true biological effects or simply sampling variation. One speculative explanation is that, in the absence of mating opportunities, males may allocate proportionally more resources to somatic growth rather than reproductive activity, although this hypothesis requires further investigation. To our knowledge, no published studies have documented this pattern, highlighting it as a potential direction for future research.

A central hypothesis in the biology of parasitic infections is the “crowding effect”, a density-dependent mechanism where high parasite numbers lead to a decrease in individual growth, size, and fecundity due to resource competition within the host intestine. While previous studies have consistently demonstrated the crowding effect (Betson et al., 2014; Elkins and Haswell-Elkins, 1989; Monzon et al., 1990; Peng and Criscione, 2012; Petkevičius et al., 1997), the magnitude of the effect in the present population remains uncertain. Our observed trend toward reduced worm size with higher worm burden was in the expected direction and the absence of a statistically significant effect may reflect limited statistical power to detect small effects in the present study, as well as the complex interplay of factors unmeasured here, or true differences in parasite crowding dynamics in this specific population. The crowding effect has been extensively documented in *Ascaris* infections in both humans and pigs (Betson et al., 2014, Peng and Criscione, 2012; Petkevičius et al., 1997), with a clear negative correlation between worm burden and female worm length (Elkins and Haswell-Elkins, 1989). Our findings, while not reaching statistical significance, align with this general principle, indicating that the crowding effect is a plausible mechanism affecting parasite development (Monzon et al., 1990).

Our second research question was about whether total worm burden was linked to the proportion of mature worms. No significant association was detected, suggesting that overall worm count may not directly influence maturation. This is consistent with the hypothesis that, although crowding may limit growth, sexual maturity is largely determined by larval development and migration within the host (Walker et al., 2009). Jung's (1954) proposal that mature worms may suppress the development of newly acquired larvae remains a possible regulatory mechanism, but our data do not provide clear support for it. These findings point to the need for further work on density-dependent effects on growth and maturation, ideally through longitudinal designs.

A core finding of this study relates to the determinants of female worm fertility, a crucial parameter in the epidemiology of ascariasis. Our results showed a significant correlation between the number, total length, and total weight of female worms and the total EPG of feces. This relationship, however, did not differ significantly between unisexual and bisexual infections. There was only a tendency toward a greater EPG output per female worm in unisexual vs bisexual infections, which would be consistent with the concept of density-dependent fecundity, or the potential “inhibition effect” on egg production. Previous work has indeed suggested that EPG per female worm tends to decrease as worm burden increases, a pattern reported even at relatively low worm counts (Hall and Holland, 2000). This finding suggests that relying solely on EPG to estimate worm burden may introduce uncertainty, particularly in settings where infection types and intensities vary.

A positive correlation between worm biometrics (length and weight) and EPG, especially in unisexual infections, is consistent with the idea that female worm size influences reproductive output. Prior studies have documented a relationship between female size and egg production, with larger worms generally producing more eggs (Walker et al., 2009). Although the associations observed here did not reach statistical significance, they appear to follow this previously described trend.

Our analysis of the male-to-female worm ratio and its association with egg fertility yielded a clearer result. We observed a negative correlation between the male-to-female worm ratio and the ratio of unfertilized-to-fertilized eggs. Rather than simply confirming that the presence of males affects fertility, which is biologically expected, this suggests a potential dose-dependent relationship, whereby higher male-to-female ratios are associated with greater rates of egg fertilization. However, this requires confirmation using larger sample sizes and an examination of potential threshold effects. While female worms can produce infertile eggs in the absence of a male, the presence of a male is essential for successful reproduction and the continuation of the life cycle. This is reported in multiple studies demonstrating the dependence of egg fertility on the presence and ratio of male worms (Hotez et al., 2008; Sinniah and Subramaniam, 1991).

We found that host age was associated with worm burden, with a decreasing trend in worm count as participant age increased. This finding is consistent with the established epidemiology of ascariasis, where peak infection prevalence and intensity are typically observed in pre-school and school-aged children (Hotez et al., 2008). This pattern is likely due to a combination of behavioral factors, such as increased exposure to contaminated soil, and the gradual development of a protective immune response with age (Cooper and Figueiredo, 2013; Hotez et al., 2008). Conversely, participant gender showed no significant association with worm count. This finding contrasts with some studies that have reported gender differences in parasitic infections, often linked to behavioral or immunological factors (Sellau et al., 2024). Additionally, our analysis confirmed the importance of household clustering, a well-documented phenomenon in ascariasis due to the shared environmental exposure and close-contact transmission within families (Incani et al., 2021).

An interesting finding is that only about 60% of the infected individuals had a full bisexual infection, in which mature eggs can be produced and easily diagnosed in routine laboratories. The remaining 40% may be missed or underdiagnosed because no eggs can be produced (only males or immature worms) or else infertile eggs may be produced and not easily diagnosed by inexperienced technicians (female-only worms). This is consistent with previous results from the same community in which fecal PCR detected significantly more *A. lumbricoides* infections than microscopy (Incani et al., 2016). PCR can overcome some limitations of routine egg-based diagnosis based on egg detection, which may fail to detect infections involving only male worms, immature worms, or infertile eggs. However, PCR may not be readily available in resource-limited settings.

In addition to the limited sample size, which represents a key limitation of this study and may have reduced the statistical power to detect significant associations, as duly acknowledged throughout the study, several other limitations should be considered when interpreting the findings. The cross-sectional design allows for the identification of associations but precludes causal inference; longitudinal data would be required to track reinfection dynamics, maturation, and fertility over time. The analysis relied on historical secondary data, which restricted the range of host-related variables available. Factors such as diet, nutritional status, and immune markers could have provided a more detailed understanding of host–parasite interactions, while genetic characterization of worms might have revealed differences in fecundity or growth not detectable from biometric measurements alone.

Data collection methods also introduce uncertainty. Although the anthelmintic treatment used has a high reported efficacy (80–100%), the assumption that all worms were expelled may not hold for every individual, potentially leading to underestimation of the worm burden. Visual classification of worm maturity based on length is a proxy measure and may not perfectly reflect true sexual maturity. Small sample sizes in several subgroups, particularly unisexual infections, further limit statistical power and may explain the lack of significance in some observed trends.

Additional methodological considerations include the absence of correction for multiple testing. Numerous statistical comparisons were conducted at an α level of 0.05, increasing the likelihood of Type I errors and raising the possibility that some apparent associations may represent false positives. Selection bias is also possible: only 128 of the 224 consenting participants (57%) provided complete fecal samples within the required timeframe. If compliance was related to infection severity, symptoms, or other unmeasured factors, the resulting sample may not fully represent the underlying population. Finally, the data were collected in 2009 but analyzed in 2025. Although the biological processes examined are unlikely to have changed, patterns of infection and transmission may have shifted due to public health interventions, potentially limiting the generalizability.

## 5 Conclusions

This exploratory study supports several established aspects of *A. lumbricoides* biology in a real-world setting. The observed patterns related to crowding, worm biometrics, and EPG, though frequently non-significant, are consistent with previous literature. The negative association between male-to-female ratio and egg fertility further supports the essential role of male worms in sustaining reproduction. Future research would benefit from larger sample sizes, longitudinal designs, and more comprehensive host-level data collections to deepen our understanding of transmission dynamics and inform control strategies.

## AI statement

During the preparation of this work the authors used Google Gemini in order to refine the clarity and flow of the English prose in selected sections. After using this tool, the authors reviewed and edited the content as needed and took full responsibility for the content of the published article.

## CRediT authorship contribution statement

**Nelson Escalona:** Writing – review & editing, Writing – original draft, Validation, Methodology, Formal analysis. **Lapo Mughini-Gras:** Writing – review & editing, Supervision, Methodology, Formal analysis. **Eva Lorenz:** Writing – review & editing, Supervision, Formal analysis. **Luis Sequera:** Writing – review & editing, Investigation. **Ivan Sequera:** Writing – review & editing, Investigation. **Ruth Serrano:** Writing – review & editing, Investigation. **Renzo Nino Incani:** Writing – review & editing, Validation, Supervision, Resources, Project administration, Investigation, Funding acquisition, Data curation, Conceptualization.

## Ethics statement

The study adhered to local ethical criteria and was approved by the Ethical Committee of the Carabobo and State Health Authority.

## Ethics declaration

Written informed consent to take part in the study and to publish the article has been obtained from all participants or their legal representatives. The privacy rights of participants have been observed.

This study included organ or tissue donors. This study includes human biological material and consent was obtained by donors, or their next of kin or legal representatives, for use in this study and for publication of the article. The samples used in this research were not sourced from executed prisoners or prisoners of conscience.

This study was performed in compliance with relevant laws, regulatory frameworks and guidelines where the research took place. This study was approved by the Ethical Committee of the Carabobo and State Health Authority (Approval No. N/A).

## Funding sources

Financial support was received from the project LOCTI-Universidad de Carabobo N° 1.235 under the sponsorship of the enterprise Nascar Autopartes, Venezuela. Project director: Renzo Nino Incani.

## Declaration of competing interest

The authors declare that they have no known competing financial interests or personal relationships that could have appeared to influence the work reported in this paper.

## Data Availability

All relevant data are within the paper.
