# Supplementary material for: Factors associated with the fertility of the helminth parasite Ascaris lumbricoides: an exploratory study in a rural Venezuelan community
Source: Food Waterborne Parasitol. 2026 Sep 23;45:e00363. doi: 10.1016/j.fawpar.2026.e00363 (PMC13635693; doi:10.1016/j.fawpar.2026.e00363)
Supplement: Supplementary file 1 [file mmc1.docx]

**SUPPLEMENTARY MATERIAL**

**Fig. S1**. *A. lumbricoides* worm load by worm sex (n = 89).

**Fig. S2.** *A. lumbricoides* egg counts (eggs per gram) by egg fertilization group.
